# A quantitative framework for stroke difficulty in table tennis beginners: multidimensional analysis and implications for training

**DOI:** 10.3389/fpsyg.2026.1806475

**Published:** 2026-04-29

**Authors:** Jiangnan Yi, Xiaoling Zhu, Wanfu Zhang, Minnan Yue, Qianlei Gu, Chun Li

**Affiliations:** 1 Department of Physical Education, University of Shanghai for Science and Technology, Shanghai, China; 2 School of Energy and Power Engineering, University of Shanghai for Science and Technology, Shanghai, China

**Keywords:** difficulty coefficient, difficulty factors, hitting difficulty, motor learning, sports training

## Abstract

**Introduction:**

This study systematically investigates the key factors influencing the difficulty of table tennis stroke tasks for beginners, aiming to establish a quantitative framework to inform evidence-based training design.

**Methods:**

Ten right-handed novice participants (mean age 21.6 ± 1.35 years) with no prior professional training completed 15 distinct ball-return tasks, each involving 20 trials. The independent variables were ball speed, frequency, height, and landing point, while the dependent variable was hitting performance. Task difficulty was quantified using a standardized difficulty coefficient formula, *p* = 1–(X/X_max_), adapted from psychometric principles.

**Results:**

Ball speed had the most profound impact on task difficulty, with performance declining sharply at 11.5 m/s (difficulty coefficient = 0.85). Increased ball frequency and spatial variability in landing points also significantly elevated difficulty. Ball height exhibited a threshold effect, where difficulty remained low within 7–44 cm but increased drastically at 170 cm. Regression analyses confirmed significant linear relationships between these parameters and the difficulty coefficient.

**Conclusion:**

The findings provide a quantitative reference for the functional task difficulty faced by beginners and offer empirical support for developing structured, evidence-based table tennis training programs. The proposed difficulty coefficient serves as a practical metric for task calibration. Future research should incorporate ball spin to enhance ecological validity.

## 1 Introduction

In research and practice, “difficulty” is defined as the degree of ease or difficulty in performing a specific task (Zhang, 2005). Based on the Limited Resource Theory, the core of task difficulty resides in the mental and physical resources consumed during task execution—the higher the difficulty, the greater the resource consumption; when the required resources exceed an individual’s capacity, the task may end in failure (Kahneman, 1973; Moray, 1967; Marteniuk, 1976). It is worth noting that task difficulty is influenced not only by the objective complexity of the task itself but also by the individual’s cognitive level, psychological state, knowledge base, and strategy selection (De Waard, 1996; Kantowitz, 1987; Parasuraman and Hancock, 2000). This has led researchers to categorize task difficulty into Nominal Task Difficulty (NTD) and Functional Task Difficulty (FTD; Sanli and Lee, 2015): Nominal Task Difficulty refers to the inherent objective difficulty of a task, while Functional Task Difficulty reflects the difficulty actually perceived by an individual and is directly related to their own skill level (Akizuki and Ohashi, 2015; Grundy et al., 2019). In the field of motor learning, improving teaching efficiency is a core goal (Ericsson et al.,1993). Guadagnoli’s Optimal Challenge Point (OCP) Theory posits that an optimal difficulty range exists for learners, within which learning effects are maximized (Guadagnoli and Lee, 2004). However, the prerequisite for applying this theory is the systematic quantification and manipulation of task difficulty. Focusing on table tennis stroke tasks, this study aims to establish a difficulty quantification method for such basic motor actions, providing empirical support for subsequent personalized training based on an individual’s optimal challenge point.

Table tennis is characterized by high variability in ball speed, spin, trajectory, and placement, making it an ideal model for studying interceptive motor skills. This inherent variability has been well documented in table tennis research. Competitive play demands rapid adaptations to continuously changing ball speeds, landing positions, and temporal rhythms (Wang, 2023; Wang, 2021; McAfee, 2009). Moreover, equipment regulations by the International Table Tennis Federation (ITTF), such as the adoption of the 40 mm ball in 2000, have explicitly aimed to moderate ball speed and spin, further highlighting the importance of these parameters for performance (Xie et al., 2008; ITTF, 2025; ITTF, 2010). Even at the beginner level, variations in ball speed, height, and landing point impose significant perceptual-motor challenges. Therefore, systematically quantifying the individual contribution of each parameter is essential for designing evidence-based training programs. Several fundamental methods exist for quantifying task difficulty. In manual aiming, Fitts’s Law links movement time to target size and distance (Fitts, 1954). Computational methods in motor control reveal neural system action planning under uncertainty (Wolpert and Ghahramani, 2000). The dual-task paradigm is widely used to assess attention demands related to functional difficulty (Abernethy, 1988; Glencross and Gould, 1979; Li and Wright, 2000; Salmoni et al., 1976). In psychometrics and educational measurement, the Pass Rate Method (P = R/N; Zhang, 2005) and the Score Rate Method (*p* = 1 − X/X_max_; National Education Examinations Authority, 1991) are classic tools. The score rate method is widely used due to its universality, for instance, in standardizing test scores and comparing performance across different test forms (Crocker and Algina, 1986).

Despite providing a foundation, these methods have limitations in sports research, particularly for interceptive actions like table tennis strokes (Gentile, 2000; Wulf and Shea, 2002). Existing studies often focus on complex motor tasks, leaving a gap in systematic difficulty assessment for beginners. While previous work shows that table tennis difficulty is affected by ball speed, spin, and landing position (Liu et al., 2012; Taghi et al., 2025; Padulo et al., 2016), the specific mechanisms by which these parameters affect beginners’ FTD remain unclear. This gap hinders the design of targeted training. Furthermore, quantifying difficulty in table tennis could inform other interceptive sports like tennis and badminton, where athletes face similar time, space, and uncertainty constraints (Laffaye et al., 2015; Phomsoupha and Laffaye, 2015; Farrow and Reid, 2010; Farrow and Reid, 2010).

Therefore, this study draws on the Score Rate Method to translate objective hitting performance into a standardized difficulty coefficient (*p* = 1 − X/X_max_). This serves a dual purpose: quantifying Functional Task Difficulty (FTD) for beginners and establishing a reference for Nominal Task Difficulty (NTD) applicable to this population. The Score Rate Method was chosen for its direct translation of performance into a standardized, relative metric, making it ideal for comparing FTD across conditions. While it does not directly measure NTD, the parameter manipulations define the objective task, and the resulting *p* values offer an empirical estimate of how novices experience that nominal difficulty. This design provides a conceptual validation that performance scores can effectively quantify functional difficulty, offering a new path for objective novice training assessment. Based on the literature, we proposed the following hypotheses:

*H*1: Ball speed significantly affects hitting difficulty—the faster the speed, the greater the difficulty;

*H*2: Ball frequency significantly affects hitting difficulty—the higher the frequency, the greater the difficulty;

*H*3: Ball height significantly affects hitting difficulty, with lower or extreme heights increasing difficulty;

*H*4: Ball landing position significantly affects hitting difficulty—the greater the variation, the greater the difficulty;

*H*5: The difficulty coefficient (P=1−X/X_max_) can effectively quantify the functional task difficulty of beginners.

## 2 Materials and methods

### 2.1 Participants

A total of 10 right-handed participants (5 male, 5 female; mean age 21.6 ± 1.35 years) with no prior formal table tennis training were recruited. All used the shakehand grip. The inclusion of only right-handed participants served as a methodological control. Recruitment began April 5, 2025, and the experiment ended April 15, 2025. All participants signed written informed consent.

The sample size (n = 10) was determined *a priori* based on a power analysis for a repeated-measures design. Assuming a large effect size (Cohen’s *f* = 0.40, equivalent to partial η^2^ ≈ 0.14), *α* = 0.05, and a conservative correlation of 0.5 among repeated measures, a sample of 10 participants provides >80% power to detect the main effects of interest (Faul et al., 2007; Cohen, 2013). This approach prioritizes internal validity for detecting theoretically predicted, large-magnitude effects in a homogeneous novice sample, consistent with methodological precedents in motor control research (Galle et al., 2017; Lohse et al., 2010).

Ethical Approval: This study was conducted in accordance with the Helsinki Declaration and approved by the Ethics Committee of University of Shanghai for Science and Technology Affiliated City East Hospital (Approval No.: IRB-AF63-V1.0; Approval Date: April 2, 2025).

### 2.2 Methods

#### 2.2.1 Experimental design

(1) To explore factors influencing table tennis return difficulty, speed, frequency, landing position, and height were identified as key factors. Spin was excluded due to equipment limitations: the ball machine could not precisely control spin independently. To ensure control, all balls were served as no-spin balls (lowest spin gear, verified by high-speed video analysis to have ~0 revolutions per second).
(2) For each factor, multiple levels were set: landing point (3 levels), speed, frequency, and height (4 levels each). See Table 1.
(3) Each task manipulated one independent variable while recording hitting performance. All participants used the backhand push technique.

**Table 1:** Experimental task design matrix.

Dimension	Variable 1	Variable 2	Variable 3	Variable 4
Speed	3.5 m/s	7.5 m/s	11.5 m/s	speed varied randomly (range: 3.5–11.5 m/s, uniform distribution)
Frequency	60 balls/min	80 balls/min	120 balls/min	frequency varied randomly (range: 60–120 balls/min, uniform distribution)
Height	7 cm	12.5 cm	44 cm	170 cm
Landing point	fixed point	two points	random points	/

For the random speed condition, the ball machine delivered speeds uniformly distributed between 3.5 and 11.5 m/s. The sequence was pre-programmed and identical for all participants to ensure standardization. Similarly, the random frequency condition used a uniform distribution between 60 and 120 balls/min.

#### 2.2.2 Quantitative method for stroke task difficulty

The difficulty coefficient was computed as: *p* = 1 – (X / X_max_), where P is the difficulty coefficient, X is the average score, and X_max_ = 20 (perfect score for 20 trials). P is positively correlated with task difficulty, ranging from 0 (easiest) to nearly 1 (most difficult).

#### 2.2.3 Experimental equipment

**Figure 1:**
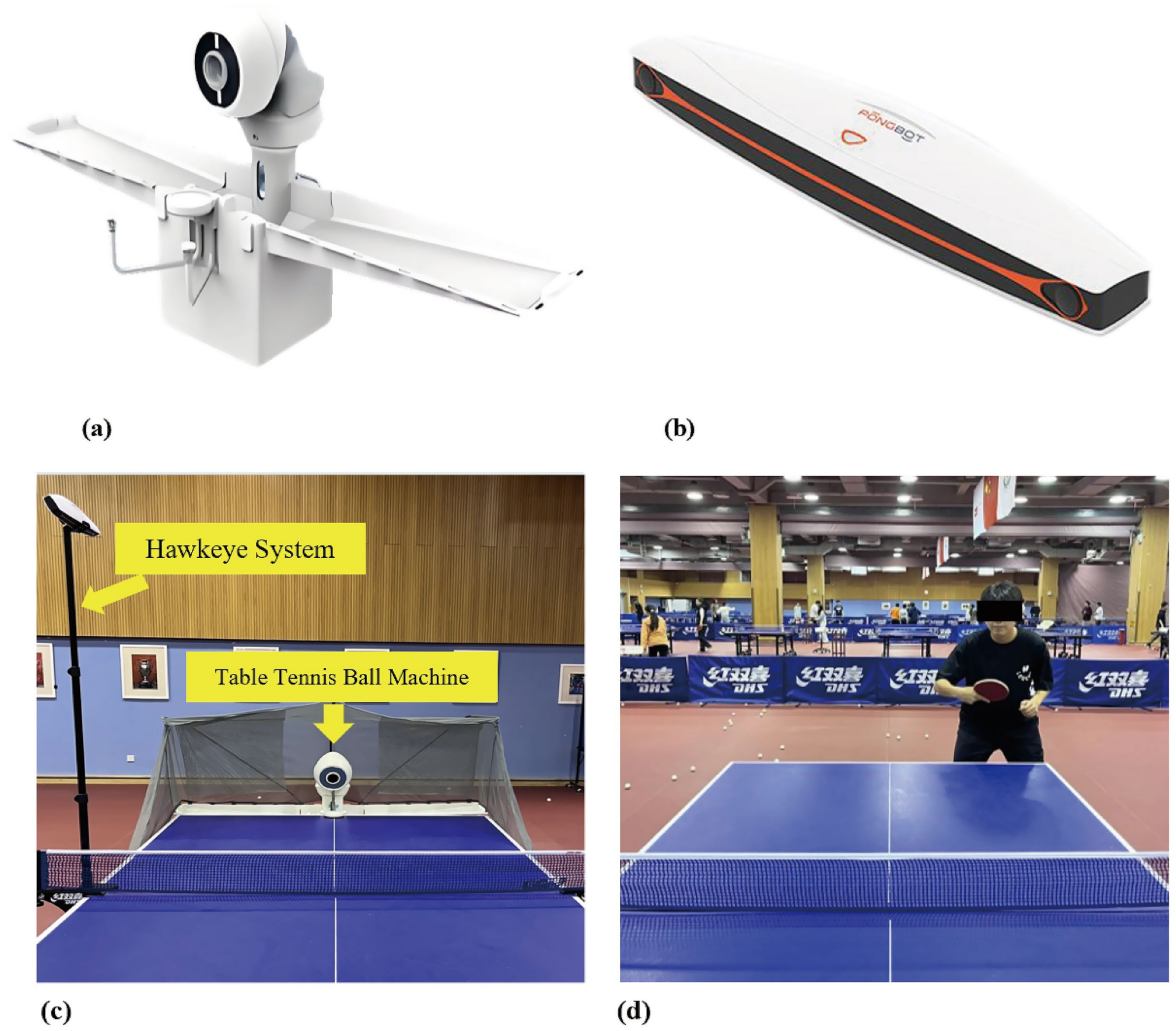
Experimental setup for ball delivery, data acquisition, and participant positioning. **(a)** The OMNI table tennis ball machine used for consistent ball delivery. **(b)** The PANGBOT-SEEKER tracking system (“Hawk-Eye”) positioned beside the net for precise measurement of ball trajectory parameters. **(c)** Overhead view showing the placement of the ball machine and the PANGBOT-SEEKER system relative to the table. **(d)** Example of a participant’s standardized standing position in the left half of the table during testing.

(1) Ball Feeding: OMNI table tennis robot (Shanghai Chuangyi Technology Co., Ltd., China).
(2) Table, Paddle, Balls: Standard Table (T1223, DHS), VISCARIA paddle (Butterfly) with Hurricane III rubber (DHS), 40 mm + 1-star Saitop balls (DHS).
(3) Calibration: The robot’s accuracy was calibrated using PANGBOT-SEEKER (Shanghai Chuangyi Technology). Margins of error: speed ±0.3 m/s, frequency ±2 balls/min, landing point radius ±5 cm.
(4) Setup: Robot placed at center of opposite table; Hawk-Eye system on both sides of the net.
(5) Participant Positioning: Standardized stance in the left half of the Table.
(6) Data Acquisition: PANGBOT-SEEKER system captured speed, landing coordinates, and net clearance height (Figure 1).

#### 2.2.4 Experimental tasks

Each task manipulated one independent variable while holding others constant at baseline (speed: 3.5 m/s; frequency: 60 balls/min; height: 8–15 cm; landing point: fixed).

**Figure 2:**
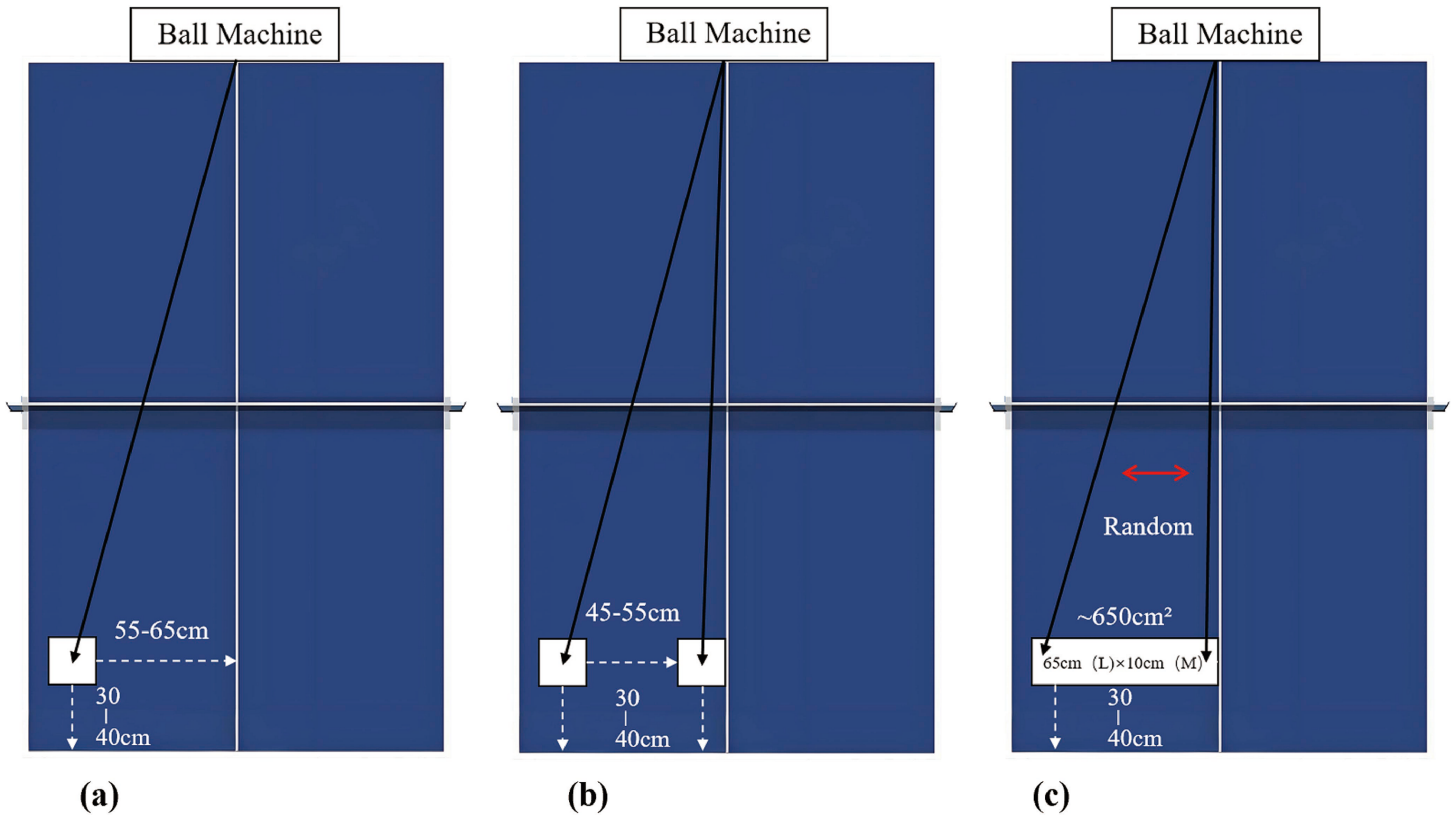
Schematic diagram of the three landing point conditions tested in the experiment: **(a)** Fixed landing point: balls delivered to a single predetermined location; **(b)** Two landing points: balls alternated between two distinct predefined locations; **(c)** Random landing points: balls delivered to pseudorandom locations within a specified rectangular area on the left half of the table.

(1) Ball Speed: Conditions: 3.5 m/s, 7.5 m/s, 11.5 m/s, random variation (3.5–11.5 m/s).
(2) Ball Frequency: Conditions: 60, 80, 120 balls/min, random variation (60–120 balls/min).
(3) Ball Height: Conditions: 7 cm, 12.5 cm, 44 cm, 170 cm. (The extreme 170 cm was chosen to represent a ‘high ball’ that challenges typical stroke mechanics, providing a contrast to the lower, more standard heights.)
(4) Landing Position: Fixed point (left half), two points (alternating between two locations 45–55 cm apart), random points (pseudorandom within a 650 cm^2^ area on the left half). See Figure 2.

#### 2.2.5 Experimental procedure

(1) Pilot Study (n = 5): Led to two refinements: a 1-min rest interval (based on heart rate recovery to <70 bpm and RPE < 10) and a participant screening criterion (≥5/10 successful returns in a screening test with speed 7.5 m/s, frequency 80 balls/min, height 7 cm, fixed point).
(2) Participant Screening: All main study participants met the screening criterion.
(3) Warm-up: Prior to the experiment, all participants completed a standardized 10-min warm-up consisting of light jogging, and dynamic stretching. Participants were tested individually in a dedicated room and could not observe other participants’ performances, eliminating observational learning effects.
(4) Pre-Test Practice: 10 practice shots per task.
(5) Task Instructions: Use backhand push; any ball landing on opponent’s table = 1 point; net balls that land on table count; misses score 0.
(6) Formal Testing: 20 attempts per task.
(7) Performance Recording: Two recorders and Hawk-Eye system. A certified coach reviewed video to confirm backhand technique compliance.
(8) Rest: 1 min between tasks.
(9) Overall Flow: Task order was fully randomized per participant to mitigate order effects. See Table 2.

**Table 2:** Flowchart of the experimental procedure.

Step 1	Step 2	Step 3	Step 4	Step 5	Step 6	Step 7
Selection test	Warm-up	Pre-test practice	Official Task 1	Break between groups 1 min	Official Test 2	Until the end

The table summarizes the sequence from participant screening and setup to task execution (with randomized order), performance recording, and inter-trial rest periods.

#### 2.2.6 Statistical analysis

A within-subjects, repeated-measures design was used. All analyses were performed with SPSS 31.0 and verified with Python 3.9.

For each factor, one-way repeated measures ANOVA was conducted. Sphericity was tested with Mauchly’s test (Greenhouse–Geisser correction applied if violated). Effect sizes are reported as partial η^2^ (0.01 = small, 0.06 = medium, 0.14 = large). Post-hoc pairwise comparisons used Bonferroni correction. Cohen’s d (0.2 = small, 0.5 = medium, 0.8 = large) is reported for pairwise contrasts.

Linear mixed models (LMM) with participant as random effect were used as a robustness check. Due to the single-factor manipulation design, formal tests of interactions were not conducted. Exploratory cross-condition comparisons are presented heuristically to generate hypotheses for future research.

## 3 Results

### 3.1 Effect of ball speed

**Figure 3:**
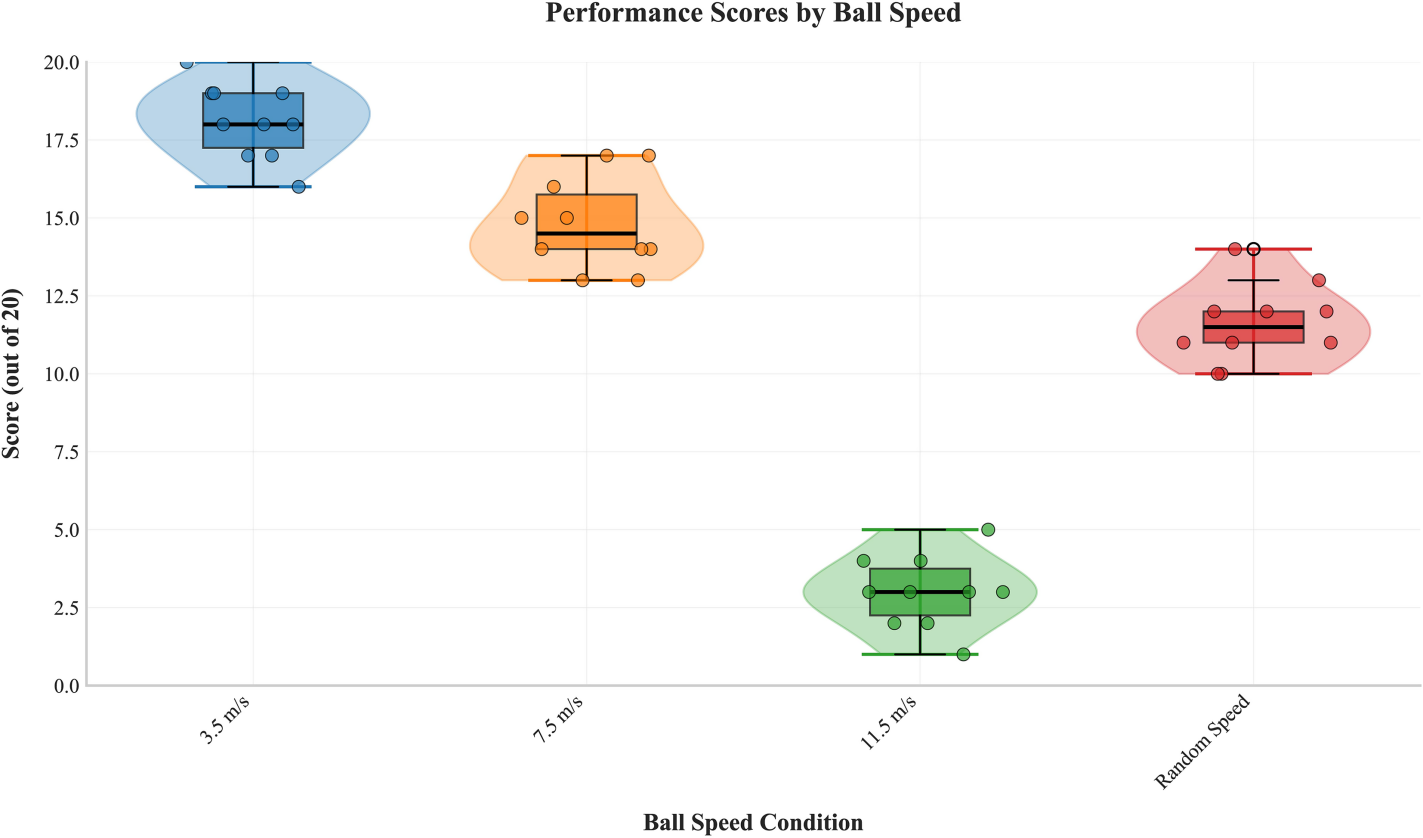
Distribution of performance scores under different ball speed conditions. Raincloud plots show the combined density distribution, boxplot, and raw data points for scores at speeds of 3.5 m/s, 7.5 m/s, 11.5 m/s, and under random speed variation.

One-way repeated measures ANOVA (Table 3) showed a significant main effect of ball speed on performance (*F*(3,27) = 284.074, *p* < 0.001, partial η^2^ = 0.969). Post-hoc comparisons (Table 4) showed a strict performance hierarchy: 3.5 m/s > 7.5 m/s > random speed > 11.5 m/s (all *p* < 0.001). The random speed condition was included to simulate match-like variability, but it introduces a confound of practice schedule (blocked vs. random). A *post-hoc* analysis excluding this condition did not alter the main conclusions regarding the significant effect of speed (*F*(2,18) = 412.5, *p* < 0.001). Figure 3 shows the distribution (Table 5).

**Table 3:** One-way repeated measures ANOVA results for tasks at different speeds.

Speed	*M* ± *SD*	*F*	*p*	Partial η^2^
3.5 m/s	18.1 ± 1.19	284.074	<0.001	0.969
7.5 m/s	14.9 ± 1.37			
11.5 m/s	3.0 ± 1.09			
speed varied randomly	11.5 ± 1.28			

Performance scores (mean ± standard deviation) and ANOVA statistics for the four ball speed conditions. The analysis indicates a statistically significant main effect of ball speed on hitting performance.

**Table 4:** Multiple comparisons of scores for tasks at different speeds (with Bonferroni correction).

(I)	(J)	Mean Difference (I-J)	95% CI	Cohen’s *d*	*p*
3.5 m/s	7.5 m/s	3.200*	[2.14, 4.26]	2.45	<0.001
	11.5 m/s	15.100*	[13.92, 16.28]	13.85	<0.001
	varied randomly	6.600*	[5.54, 7.66]	5.16	<0.001
7.5 m/s	3.5 m/s	−3.200*	[−4.26, −2.14]	−2.45	<0.001
	11.5 m/s	11.900*	[10.72, 13.08]	10.91	<0.001
	varied randomly	3.400*	[2.34, 4.46]	2.66	<0.001
11.5 m/s	3.5 m/s	−15.100*	[−16.28, −13.92]	−13.85	<0.001
	7.5 m/s	−11.900*	[−13.08, −10.72]	−10.91	<0.001
	varied randomly	−8.500*	[−9.56, −7.44]	−7.80	<0.001
varied randomly	3.5 m/s	−6.600*	[−7.66, −5.54]	−5.16	<0.001
	7.5 m/s	−3.400*	[−4.46, −2.34]	−2.66	<0.001
	11.5 m/s	8.500*	[7.44, 9.56]	7.80	<0.001

*indicates significant difference after Bonferroni correction (*p* < 0.05). Cohen’s d interpretation: small (0.2), medium (0.5), large (0.8).

**Table 5:** One-way repeated measures ANOVA results for tasks at different frequencies.

Frequency	*M* ± *SD*	*F*	*p*	partial η^2^
60 balls/min	17.7 ± 1.15	34.551	<0.001	0.793
80 balls/min	15.4 ± 0.96			
120 balls/min	10.9 ± 2.62			
Varied randomly	13.3 ± 2.62			

Performance scores (mean ± standard deviation) and ANOVA statistics for the four ball frequency conditions. The results show a significant main effect of frequency on task performance.

### 3.2 Effect of frequency

**Figure 4:**
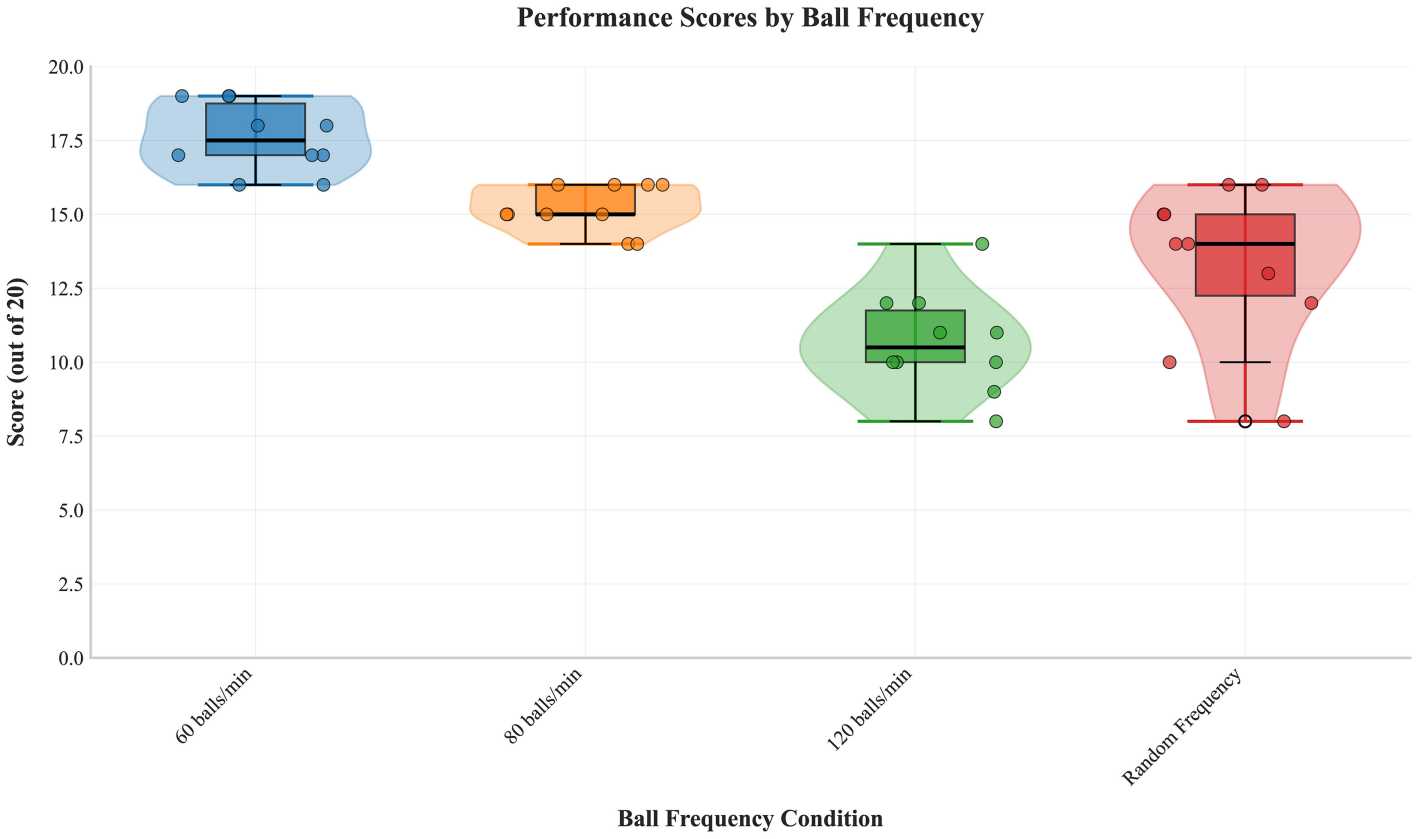
Performance score distribution across varied ball frequencies. Raincloud plots visualize the score distributions for frequencies of 60, 80, 120 balls/min and under random frequency variation, highlighting changes in central tendency and variability.

A significant main effect was found (*F*(3,27) = 34.551, *p* < 0.001, partial η^2^ = 0.793). Post-hoc tests (Table 6) showed 60 > 80 > 120 balls/min (all *p* < 0.001). The random frequency condition was not significantly different from 80 balls/min (*p* = 0.162) but showed large effect sizes (Cohen’s *d* = 0.99–1.13) compared to others, suggesting meaningful differences. Figure 4 shows distributions (Table 7).

**Table 6:** Multiple comparisons of scores for tasks at different frequencies (with Bonferroni correction).

(I)	(J)	Mean difference (I-J)	95% CI	Cohen’s *d*	*p*
60 balls/min	80balls/min	2.300*	[1.45, 3.15]	2.18	<0.001
	120balls/min	6.800*	[4.75, 8.85]	3.21	<0.001
	varied randomly	4.400*	[2.85, 5.95]	2.08	<0.001
80 balls/min	60balls/min	−2.300*	[−3.15, −1.45]	−2.18	<0.001
	120balls/min	4.500*	[2.45, 6.55]	2.12	<0.001
	varied randomly	2.100*	[0.55, 3.65]	0.99	0.027
120 balls/min	60balls/min	−6.800*	[−8.85, −4.75]	−3.21	<0.001
	80balls/min	−4.500*	[−6.55, −2.45]	−2.12	<0.001
	varied randomly	−2.400*	[−4.25, −0.55]	−1.13	0.013
Varied randomly	60balls/min	−4.400*	[−5.95, −2.85]	−2.08	<0.001
	80balls/min	−2.100*	[−3.65, −0.55]	−0.99	0.027
	120balls/min	2.400*	[0.55, 4.25]	1.13	0.013

*indicates significant difference after Bonferroni correction (*p* < 0.05). Cohen’s d interpretation: small (0.2), medium (0.5), large (0.8).

**Table 7:** One-way repeated measures ANOVA results for tasks at different heights.

Height	*M* ± *SD*	*F*	*p*	Partial η^2^
7 cm	18.8 ± 1.22	143.793	<0.001	0.941
12.5 cm	18.3 ± 1.15			
44 cm	17.9 ± 1.44			
170 cm	9.10 ± 1.37			

Performance scores (mean ± standard deviation) and ANOVA statistics for the four ball height conditions. The analysis reveals a significant overall effect, primarily driven by the extreme height condition.

### 3.3 Effect of height

**Figure 5:**
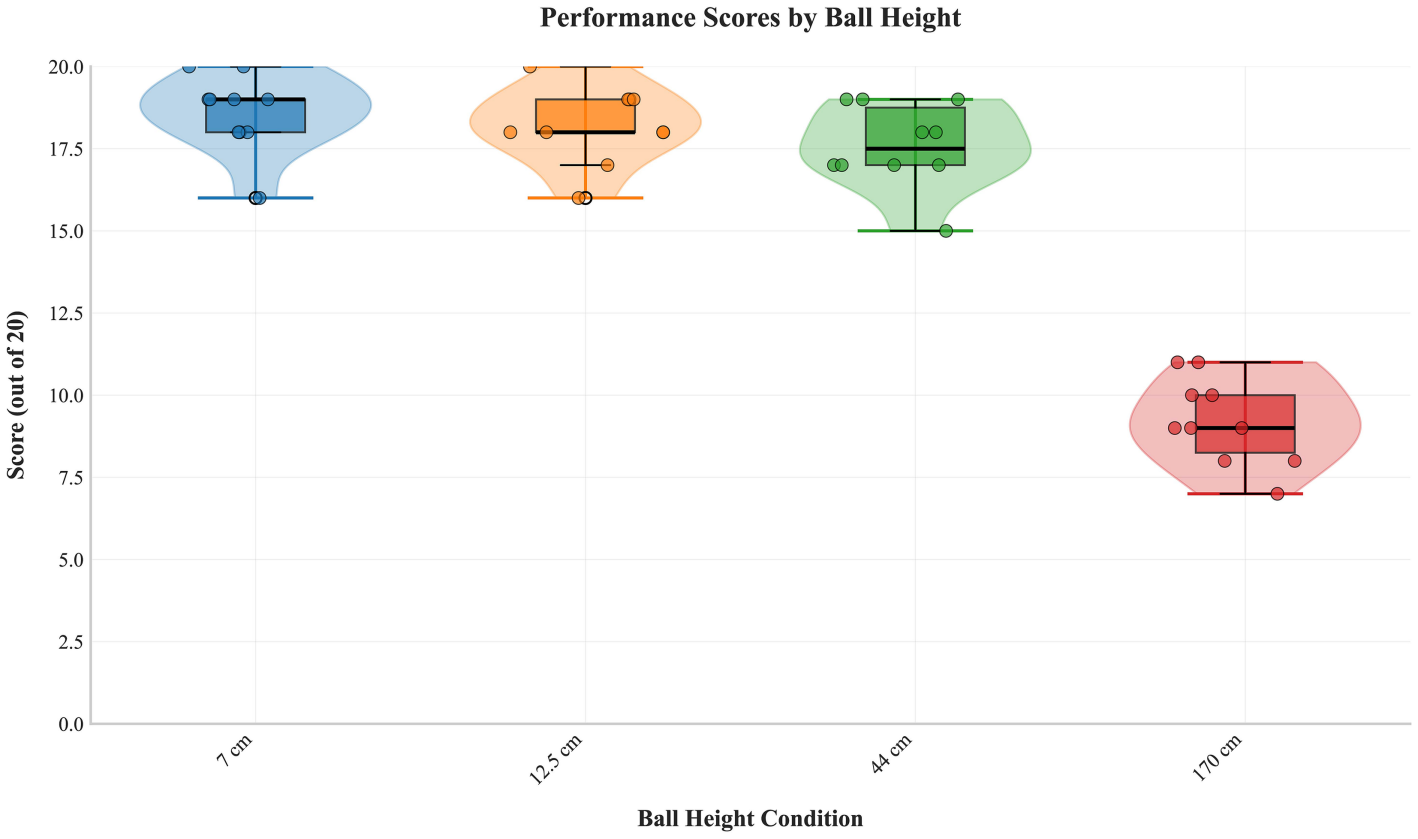
Distribution of hitting scores as a function of ball height. Raincloud plots compare performance distributions across four net clearance heights (7 cm, 12.5 cm, 44 cm, 170 cm), illustrating the threshold effect where performance collapses only at the extreme height.

A significant main effect was found (*F*(3,27) = 143.793, *p* < 0.001, partial η^2^ = 0.941). Post-hoc tests (Table 8) revealed a threshold effect: no significant differences among 7 cm, 12.5 cm, and 44 cm (all *p* > 0.05), but all were significantly better than 170 cm (*p* < 0.001). Figure 5 illustrates this threshold (Table 9).

**Table 8:** Multiple comparisons of scores for tasks at different heights (with Bonferroni correction).

(I)	(J)	Mean difference (I-J)	95% CI	Cohen’s *d*	*p*
7 cm	12.5 cm	0.500	[−0.47, 1.47]	0.43	0.244
	44 cm	0.900	[−0.07, 1.87]	0.67	0.068
	170 cm	9.700*	[8.73, 10.67]	7.00	<0.001
12.5 cm	7 cm	−0.500	[−1.47, 0.47]	−0.43	0.244
	44 cm	0.400	[−0.57, 1.37]	0.29	0.509
	170 cm	9.200*	[8.23, 10.17]	6.64	<0.001
44 cm	7 cm	−0.900	[−1.87, 0.07]	−0.67	0.068
	12.5 cm	−0.400	[−1.37, 0.57]	−0.29	0.509
	170 cm	8.800*	[7.83, 9.77]	6.35	<0.001
170 cm	7 cm	−9.700*	[−10.67, −8.73]	−7.00	<0.001
	12.5 cm	−9.200*	[−10.17, −8.23]	−6.64	<0.001
	44 cm	−8.800*	[−9.77, −7.83]	−6.35	<0.001

*indicates significant difference after Bonferroni correction (*p* < 0.05). Cohen’s d interpretation: small (0.2), medium (0.5), large (0.8).

**Table 9:** One-way repeated measures ANOVA results for tasks with different landing points.

Landing spot	*M* ± *SD*	*F*	*p*	Partial η^2^
Fixed point	18.4 ± 0.97	69.876	<0.001	0.886
Two points	14.6 ± 1.51			
Random points	12.4 ± 1.58			

Performance scores (mean ± standard deviation) and ANOVA statistics for the three levels of landing point predictability (fixed, two points, random). A significant main effect of spatial predictability was found.

### 3.4 Effect of landing point

**Figure 6:**
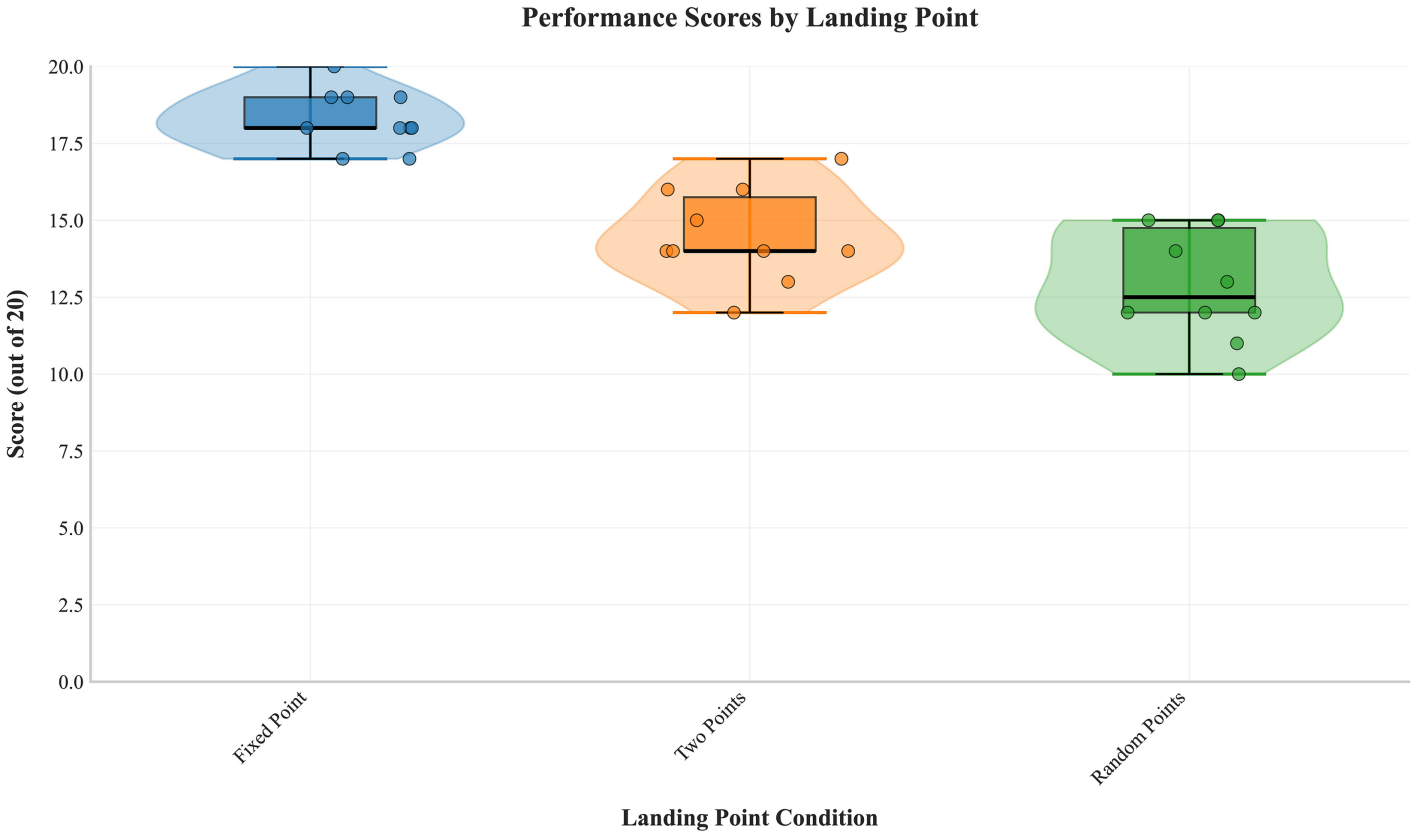
Performance distribution for tasks with different ball landing points. Raincloud plots depict the score distributions for fixed-point, two-point, and random landing point conditions, demonstrating increasing difficulty and variability with greater spatial uncertainty.

A significant main effect was found (*F*(2,18) = 69.876, *p* < 0.001, partial η^2^ = 0.886). Pairwise comparisons (Table 10) showed fixed point > two points > random points (all *p* < 0.01). Figure 6 shows distributions.

**Table 10:** Multiple comparisons of scores for tasks with different landing points (with Bonferroni correction).

(I)	(J)	Mean difference (I-J)	95% CI	Cohen’s *d*	*p*
Fixed point	two points	3.800*	[2.85, 4.75]	2.88	<0.001
	random points	6.000*	[4.80, 7.20]	3.59	<0.001
Two points	fixed point	−3.800*	[−4.75, −2.85]	−2.88	<0.001
	random points	2.200*	[0.94, 3.46]	1.25	0.006
Random points	fixed point	−6.000*	[−7.20, −4.80]	−3.59	<0.001
	two points	−2.200*	[−3.46, −0.94]	−1.25	0.006

*indicates significant difference after Bonferroni correction (*p* < 0.05). Cohen’s d interpretation: small (0.2), medium (0.5), large (0.8).

### 3.5 Difficulty quantification and regression

**Figure 7:**
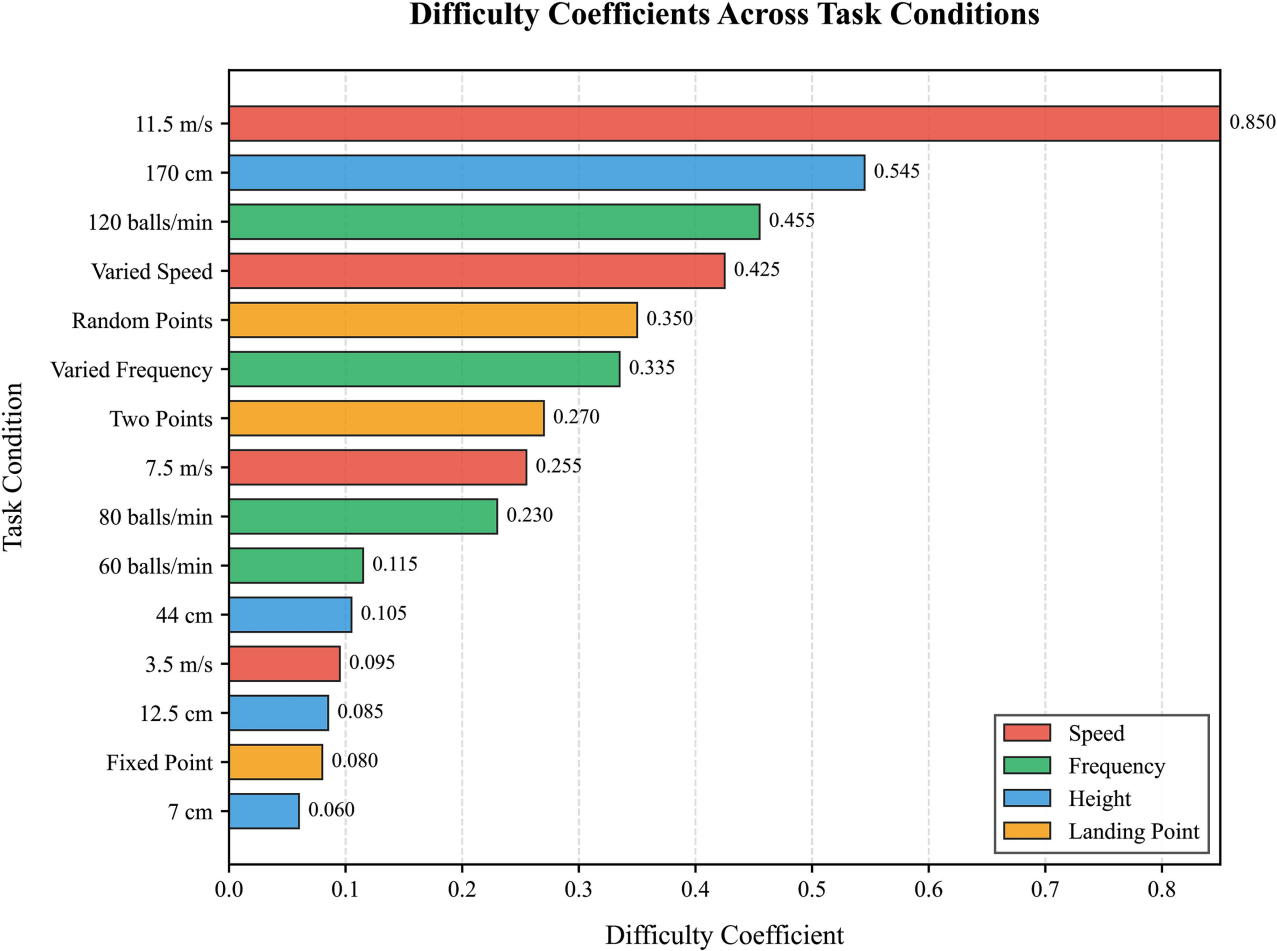
Difficulty coefficients for all 15 experimental task conditions. A bar chart ranks the calculated difficulty coefficient (*P*) for each of the 15 unique task conditions, visually identifying the most challenging parameter combinations.

Difficulty coefficients for all 15 conditions are shown in Figure 7. The highest difficulty was for 11.5 m/s speed (*p* = 0.85), followed by 170 cm height (*p* = 0.58) and 120 balls/min frequency (*p* = 0.455).

**Figure 8:**
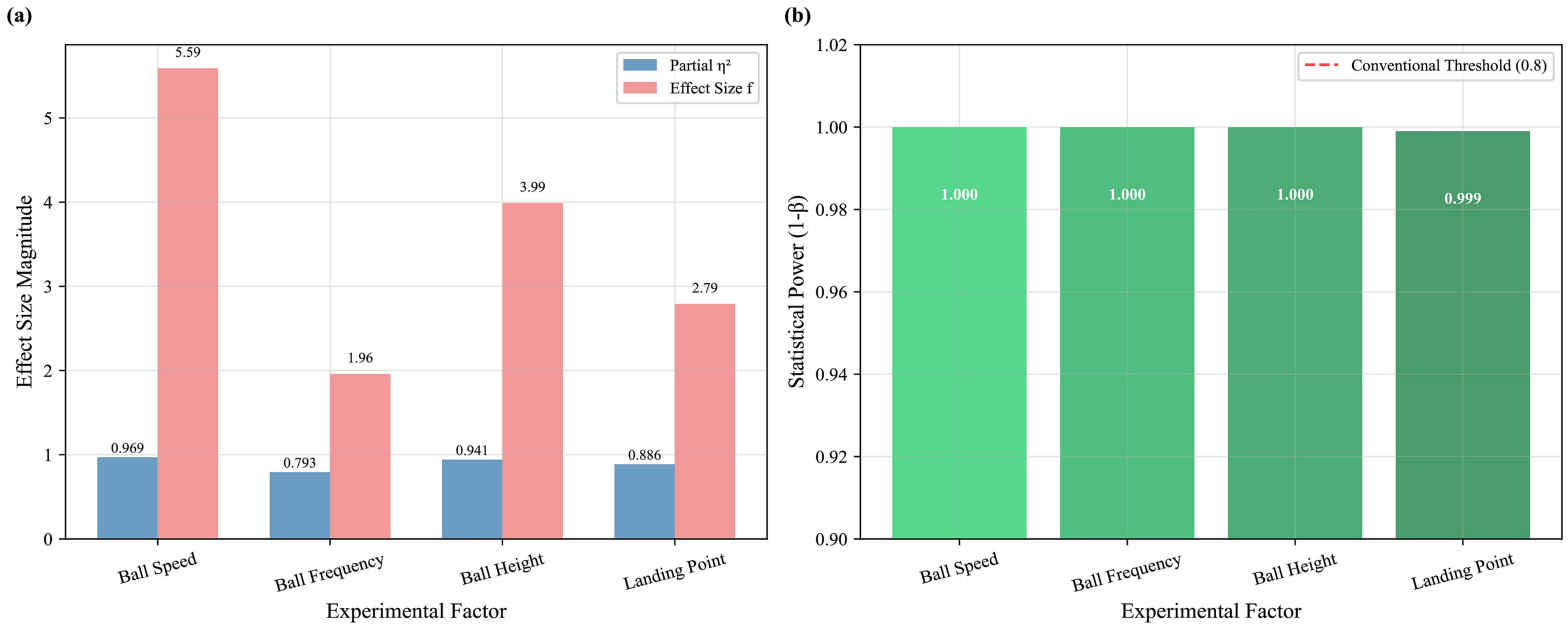
Effect sizes and statistical power of the experimental factors. **(a)** Bar chart comparing the partial η^2^ and effect size *f* for the four main factors (speed, frequency, height, landing point). **(b)** Results of the post-hoc power analysis, showing observed statistical power for each factor against the conventional 0.8 threshold.

Post-hoc power analysis (Figure 8) showed power >0.99 for all main factors, exceeding the 0.80 threshold (Lakens, 2013).

Linear regression was conducted separately for each factor (speed, frequency, height) predicting the difficulty coefficient. Results (Table 11) showed significant linear relationships: Speed explained 88.2% of variance (*β* = 0.939, *p* < 0.01), frequency explained 83.5% (*β* = 0.060, *p* < 0.01), and height explained 85.0% (*β* = 0.260, *p* < 0.01). Multicollinearity was not assessed as separate models were used.

**Table 11:** Regression analysis of the factors affecting task difficulty.

Variate	*β*	*R^2^*	*t*	*p*	*F*
Speed	0.939	0.882	14.487	<0.01	209.869
Frequency	0.060	0.835	11.888	<0.01	141.334
Height	0.260	0.850	14.648	<0.01	214.565

All regression models were statistically significant (*p* < 0.01). β values represent standardized coefficients.

### 3.6 Heuristic analysis of potential interaction patterns

**Figure 9:**
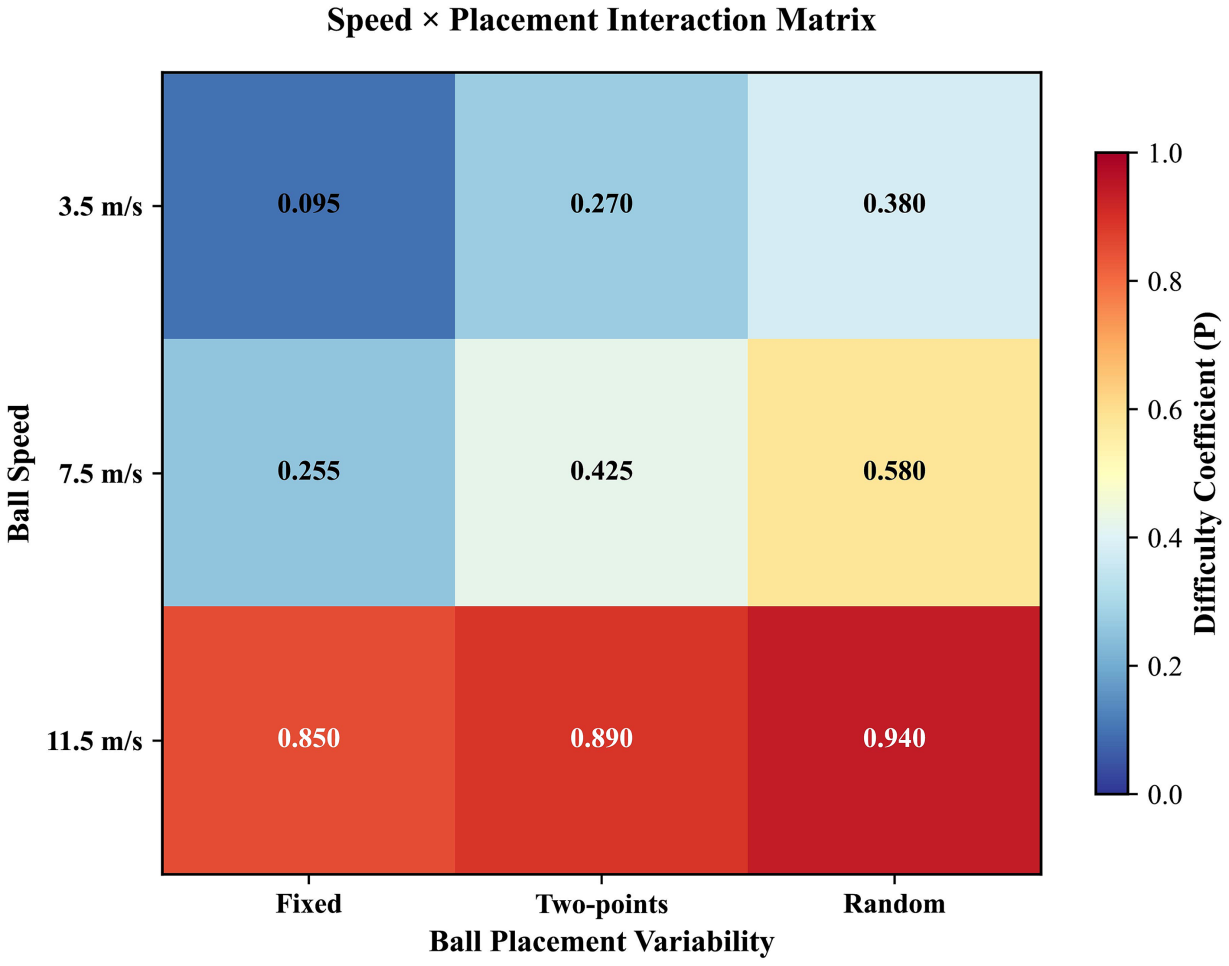
Interactive effects of ball speed and placement variability on task difficulty. A heatmap visualizes the difficulty coefficient across combinations of ball speed levels and landing point conditions, suggesting a non-linear increase in challenge when high speed coincides with spatial uncertainty.

**Figure 10:**
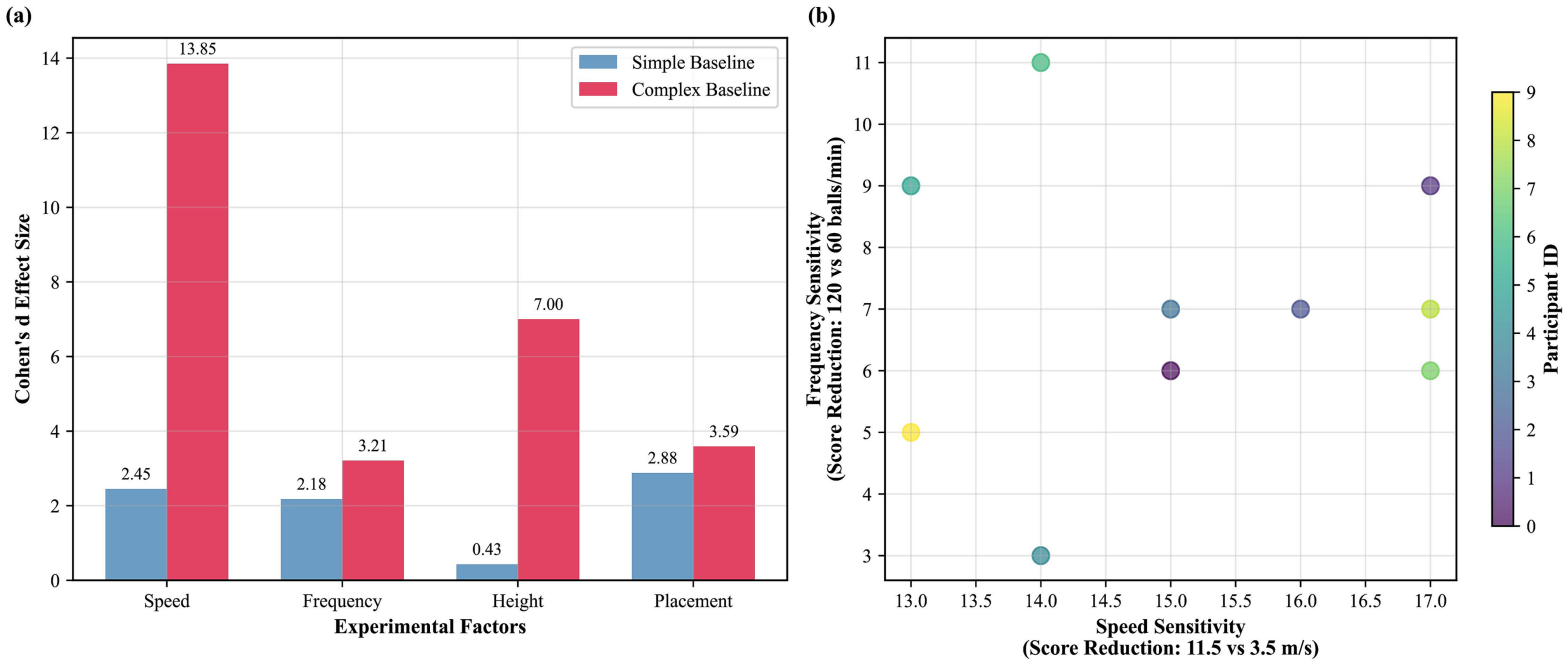
Modulation of factor effects by task complexity and individual sensitivity patterns. **(a)** Comparison of effect sizes (Cohen’s d) for key factors under simple versus complex baseline conditions. **(b)** Scatter plot showing individual participants’ sensitivity to speed versus frequency challenges, revealing heterogeneous response patterns.

**Figure 11:**
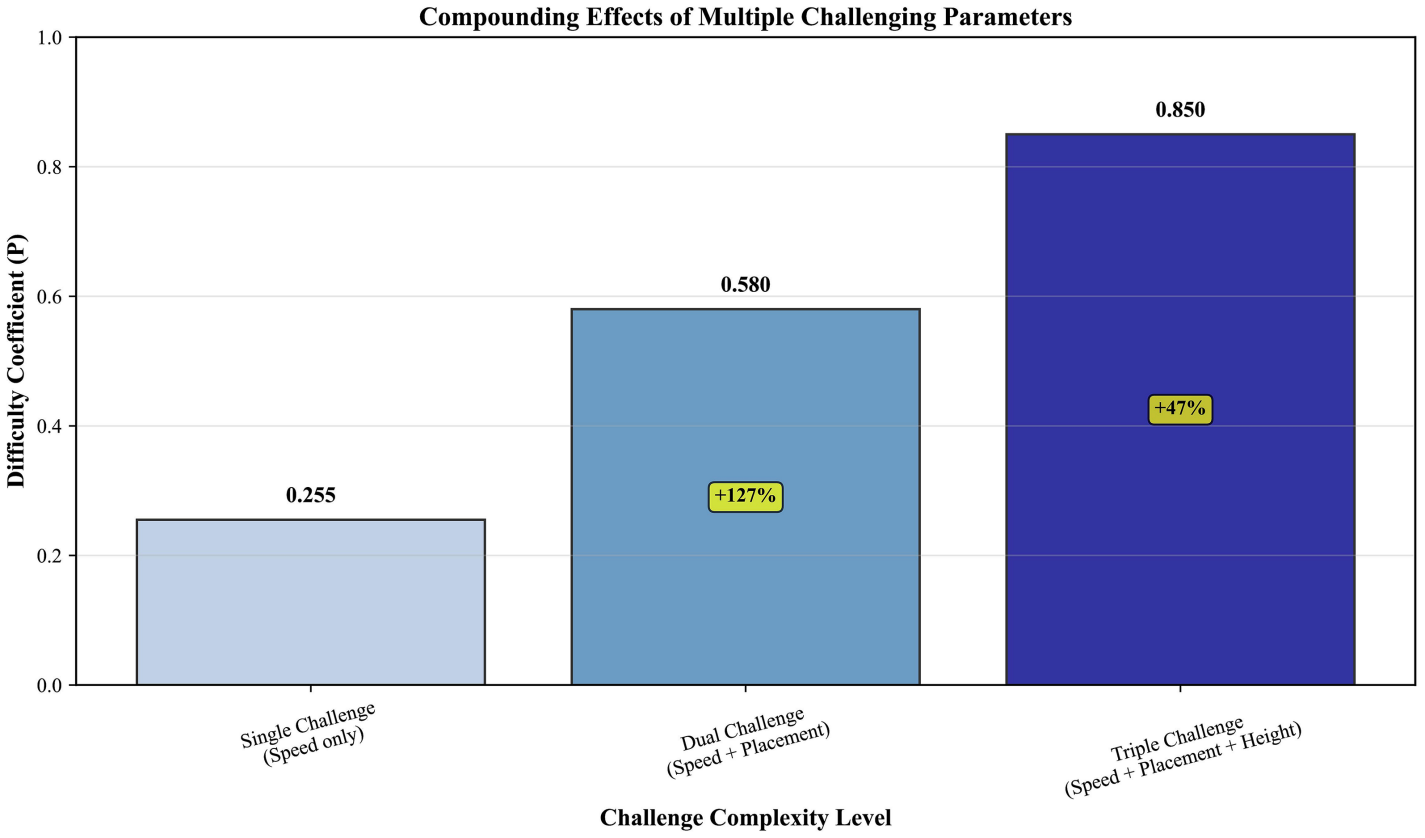
Compounding effects of multiple challenging parameters on task difficulty. A bar chart quantifies the progressive increase in the difficulty coefficient as challenging parameters (speed, placement, height) are combined, demonstrating a supra-additive effect.

**Figure 12:**
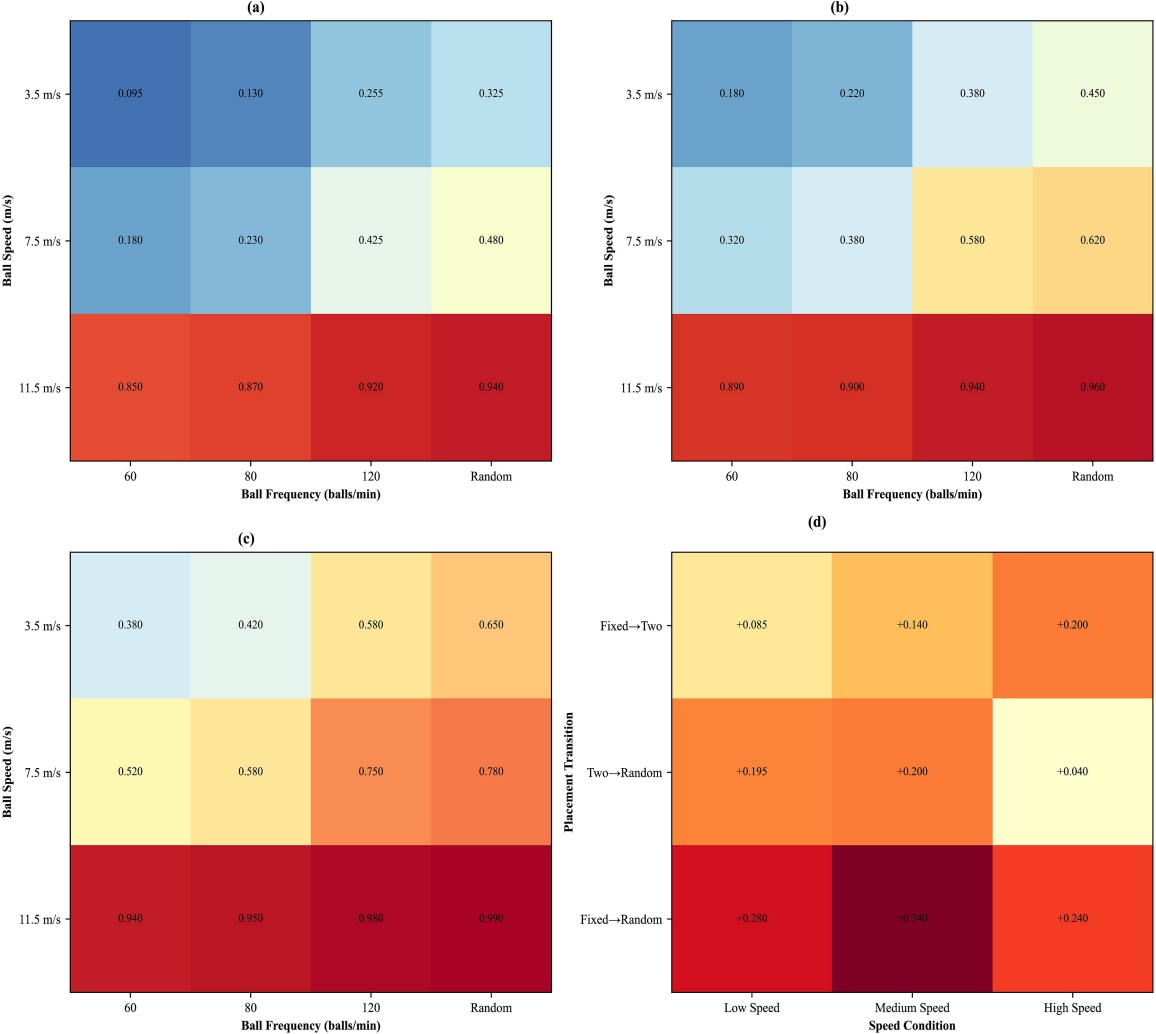
Multidimensional analysis of parameter interactions across placement conditions. **(a–c)** Heatmaps showing the speed-frequency interaction under fixed, two-point, and random placement conditions, respectively. **(d)** Bar chart showing the incremental difficulty added by placement variability at different speed levels.

**Figure 13:**
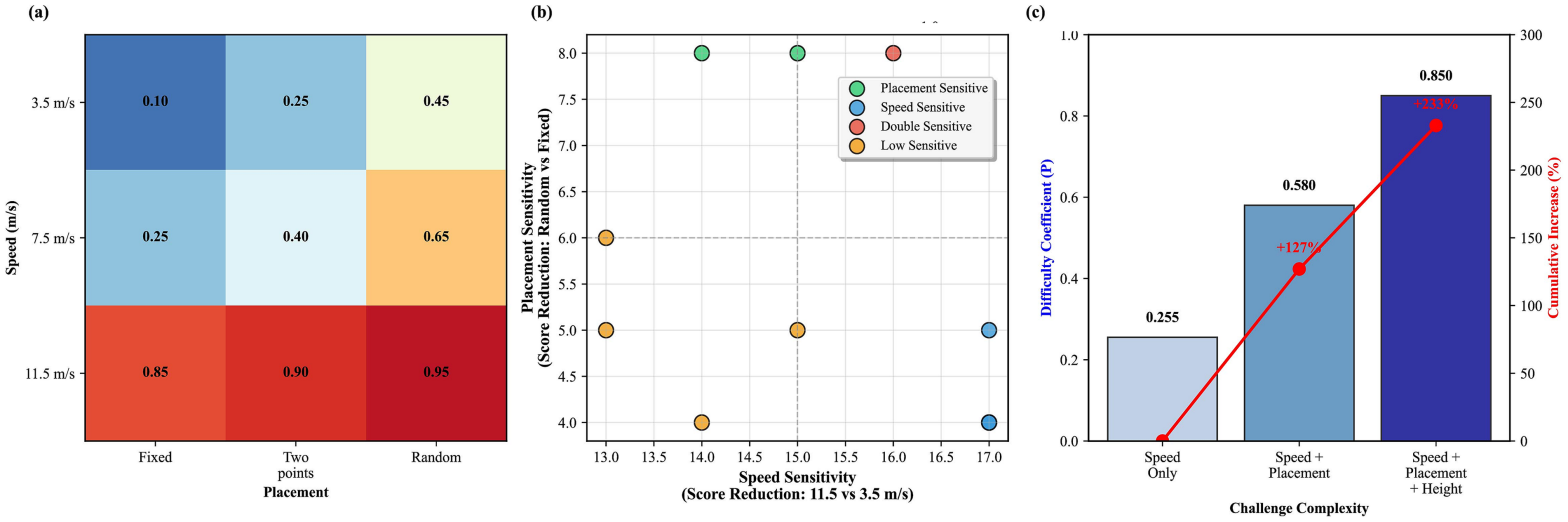
Integrated analysis of interaction patterns and individual differences. **(a)** Visual summary of compound challenge effects. **(b)** Clustering of participants based on their sensitivity profiles to speed and placement challenges. **(c)** Quantification of non-linear difficulty accumulation.

**Figure 14:**
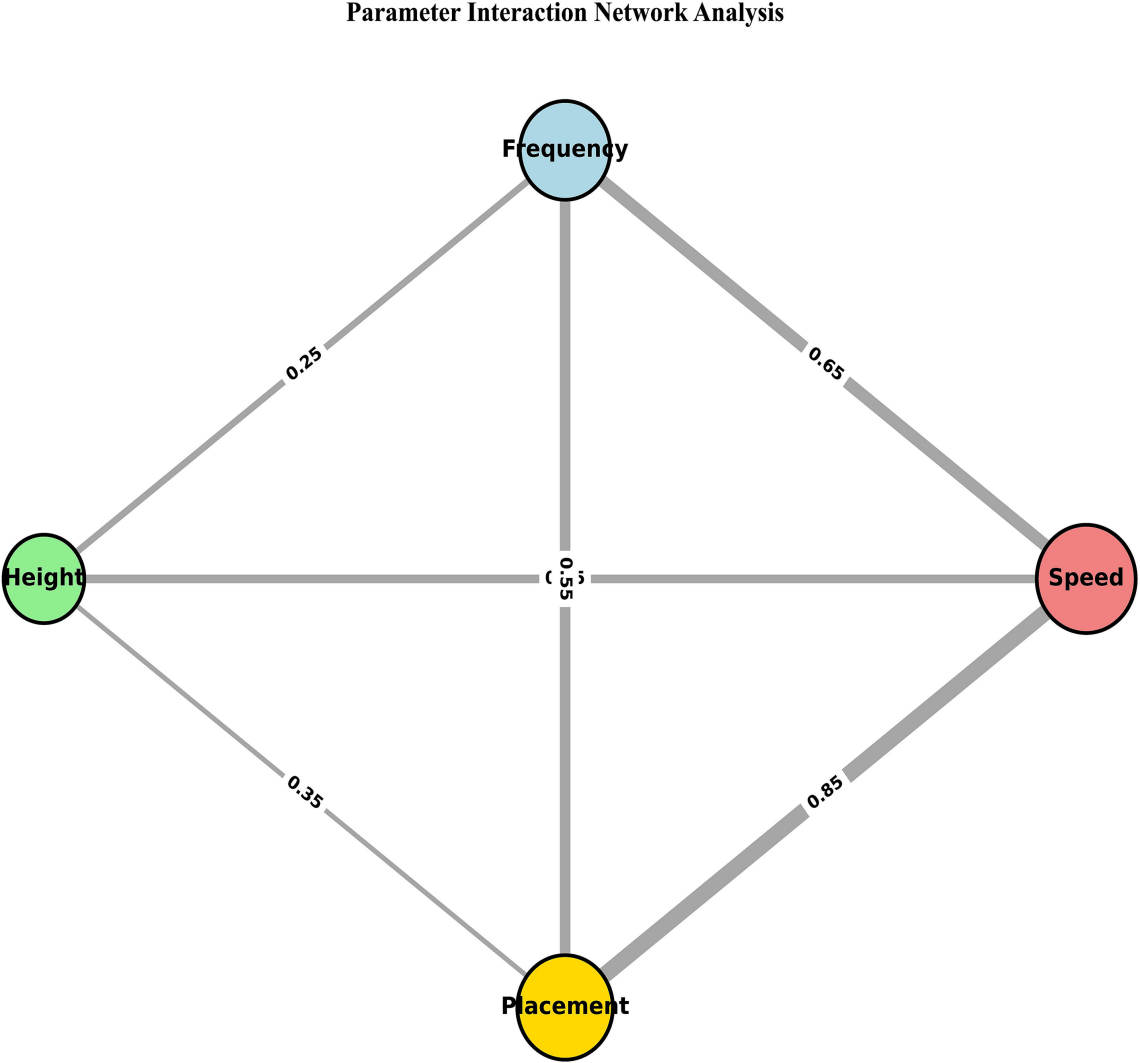
Network visualization of parameter interactions in table tennis task difficulty. A network graph where nodes represent experimental parameters (speed, frequency, height, landing point) and edge weights represent the inferred strength of their interactive effects on task difficulty.

Due to the single-factor design, formal interaction tests are not possible. The following cross-condition comparisons are exploratory and intended only to generate hypotheses for future research. Figures 9–14 illustrate patterns suggesting that combining challenging parameters (e.g., high speed + random placement) may produce difficulty exceeding additive effects. For example, Figure 11 shows a 127% increase from single to dual challenges, and a further 47% to triple challenges. These patterns, while preliminary, indicate that future factorial designs are warranted.

## 4 Discussion

### 4.1 Principal findings and theoretical integration

This study provides quantitative evidence that ball speed, frequency, landing point, and height shape FTD for beginners. The results support H1, H2, H4, H5, and partially refine H3. Ball speed had the strongest effect (H1 supported). Frequency significantly increased difficulty (H2 supported). Height showed a threshold effect, not a simple linear relationship (H3 refined). Landing point variability systematically increased difficulty (H4 supported). The difficulty coefficient successfully ranked conditions (H5 supported). These findings align with Limited Resource Theory (Kahneman, 1973; Moray, 1967; Marteniuk, 1976) and Reaction Time Theories, and lay groundwork for applying OCP theory (Guadagnoli and Lee, 2004).

### 4.2 Key parameters

Ball Speed and Frequency: Speed was the dominant factor, with catastrophic drop at 11.5 m/s. Random speed presented intermediate difficulty. High frequency (120 balls/min) imposed rhythmic pressure. The random frequency condition induced high performance variability, suggesting added cognitive load for anticipation (Bootsma et al., 2018; Abernethy, 1990; Sternad, 2018).

Ball Height: Exhibited a threshold effect. Performance was stable across 7–44 cm (an “optimal striking zone”) but collapsed at 170 cm, forcing unstable postures and disrupting coordination (Schmidt et al., 2018). This aligns with ecological dynamics (Button et al., 2021).

Landing Point: Systematic increase in spatial unpredictability (fixed → two-point → random) elevated difficulty by increasing cognitive demands for anticipatory positioning (Davids et al., 2008).

Interpretation through Cognitive and Neuromuscular Load: High speed and frequency primarily escalate cognitive demands (Limited Resource Theory). Randomness amplifies cognitive load. The 170 cm height pushes beginners toward neuromuscular limitations, requiring rapid postural adjustments beyond their coordination capabilities (Horak, 2006; Ting and McKay, 2007; Kleim and Jones, 2008). High-speed and random conditions demand rapid force generation, taxing rate of force development (RFD) in novices (Folland and Williams, 2007; Aagaard et al., 2002; Bishop et al., 2025).

### 4.3 Heuristic interaction patterns

The exploratory cross-condition comparisons suggest that combining challenges (e.g., speed + placement) might create compound difficulty (Figures 9–14). However, these findings are hypothesis-generating only. Potential mechanisms could include cognitive-motor resource competition, threshold effects, and individual differences in vulnerability. These patterns await confirmation via dedicated factorial designs.

### 4.4 A quantified framework for training

**Figure 15:**
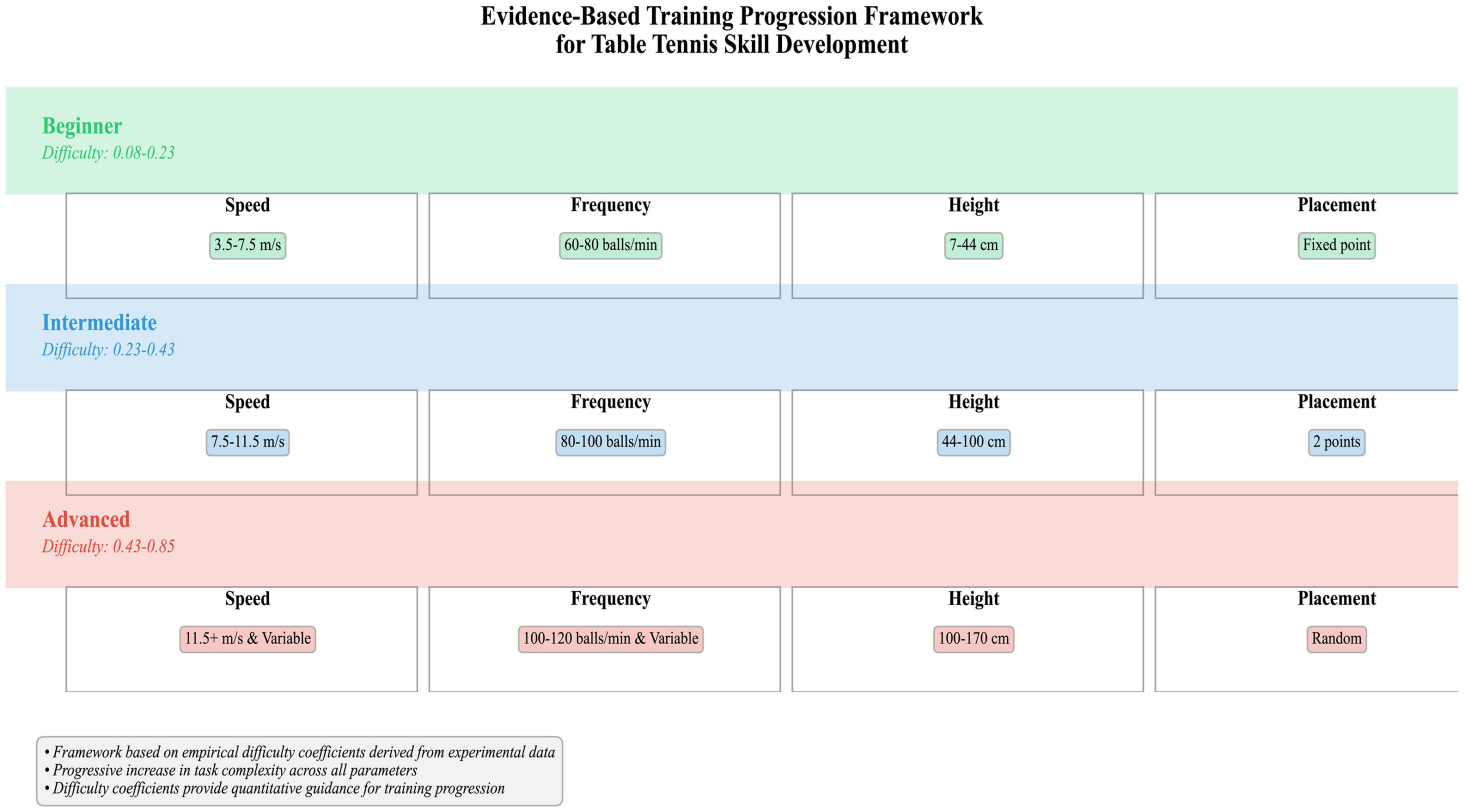
Proposed training progression framework based on quantitative difficulty coefficients. A staged model recommending parameter ranges (speed, frequency, height, placement variability) for beginner, intermediate, and advanced training, mapped to corresponding difficulty coefficient ranges.

Based on the difficulty coefficients, a progressive training framework is proposed (Figure 15):

(1) Beginner (*p* = 0.08–0.23): Fixed points, 3.5–7.5 m/s, 60–80 balls/min, height 7–44 cm.
(2) Intermediate (*p* = 0.23–0.43): Two-point placements, 7.5–11.5 m/s, 80–100 balls/min, height 44–100 cm.
(3) Advanced (*p* = 0.43–0.85): Random placements, >11.5 m/s (variable), 100–120 balls/min, height 100–170 cm.

### 4.5 Broader implications

The principles may transfer to other interceptive sports (tennis, badminton) and could inform digital training systems (e.g., apps adjusting ball machine parameters) and rehabilitation protocols (progressive challenge) (Kwakkel et al., 2019; Baladaniya and Baldania, 2024).

### 4.6 Limitations and future directions

Methodological considerations: A central contribution of this study is the proposal and application of a quantitative difficulty coefficient for motor tasks, adapted from psychometrics. The coefficient *p* = 1 − X/X_max_ possesses high face validity for quantifying Functional Task Difficulty (FTD) in this context, as it directly translates objective performance (X) into a standardized index of challenge. However, we acknowledge that this is a novel application in motor performance research. The measure’s construct validity would be strengthened by correlating it with other indices of difficulty in future studies, such as participants’ subjective ratings of mental and physical effort (e.g., NASA-TLX), or physiological measures of arousal. The use of a fixed X_max_ = 20 based on the task design is a strength for standardization but also a limitation, as it does not account for the theoretical possibility of a performance ceiling that might be lower than 20 for even the simplest condition for absolute beginners. Despite these considerations, the coefficient successfully served its primary purpose: to provide a clear, ordinal ranking of task difficulty that aligns well with intuitive expectations and theoretical predictions, establishing a proof-of-concept for its utility in sports science.

Sample Size and Generalizability: The small, homogeneous sample (n = 10, all right-handed beginners) limits generalizability to experts or other populations. While the within-subjects design and *a priori* power analysis support internal validity, future research with larger, more diverse samples is needed.

Exclusion of Spin: Spin was excluded due to equipment limitations, reducing ecological validity. Future studies should use advanced ball machines or human feeders to manipulate spin.

Single-Factor Design: The single-factor manipulation precludes formal interaction tests. The exploratory interaction patterns reported are heuristic. Future factorial designs (e.g., 3×3) are required to test interactions.

Lack of Kinematic Data: Performance accuracy data do not reveal underlying motor control adjustments. Future work should incorporate motion capture or wearable sensors.

Inference of Cognitive Mechanisms: Conclusions about cognitive load are indirect. Future research should use EEG, eye-tracking, or dual-task paradigms for direct evidence.

## 5 Conclusion

This study establishes a preliminary quantitative framework for stroke difficulty in table tennis beginners. Key findings:

(1) Ball speed is the dominant factor, with a non-linear difficulty increase.
(2) Frequency and landing point variability significantly elevate difficulty.
(3) Ball height shows a threshold effect, with an optimal striking zone (7–44 cm).
(4) The difficulty coefficient (*p* = 1-X/X_max_) is a valid metric for quantifying FTD.
(5) The framework links theory to practice and may inform digital training innovation. Future research should incorporate spin, test interactions factorially, and validate across skill levels.

## Data Availability

The data analyzed in this study is subject to the following licenses/restrictions: the datasets used and/or analyzed during the current study are available from JY upon reasonable request, yijiangnan@usst.edu.cn. Requests to access these datasets should be directed to yijiangnan@usst.edu.cn.

## References

[ref1] Aagaard P. Simonsen E. B. Andersen J. L. Magnusson P. Dyhre-Poulsen P. (2002). Increased rate of force development and neural drive of human skeletal muscle following resistance training. J. Appl. Physiol. 93, 1318–1326. doi: 10.1152/japplphysiol.00283.2002, 12235031

[ref2] Abernethy B. (1988). Dual-task methodology and motor skills research: some applications and methodological constraints. J. Hum. Mov. Stud. 14, 101–132.

[ref3] Abernethy B. (1990). Expertise, visual search, and information pick-up in squash. Perception 19, 63–77. 2336337 10.1068/p190063

[ref4] Akizuki K. Ohashi Y. (2015). Measurement of functional task difficulty during motor learning: what level of difficulty corresponds to the optimal challenge point? Hum. Mov. Sci. 43, 107–117. doi: 10.1016/j.humov.2015.07.007, 26253223

[ref5] Baladaniya M. Baldania S. (2024). Emerging trends in physical therapy for stroke rehabilitation: a comprehensive review. Int. J. Sci. Res. 13, 479–485. doi: 10.21275/SR24226223922

[ref6] Bishop D. J. Beck B. E. L. Biddle S. J. Denay K. L. Ferri A. Gibala M. J. . (2025). Physical activity and exercise intensity terminology: a joint American College of Sports Medicine (ACSM) expert statement and exercise and sport science Australia (ESSA) consensus statement. J. Sci. Med. Sport 57, 2599–2613. doi: 10.1249/MSS.0000000000003795, 41085254

[ref7] Bootsma J. M. Hortobágyi T. Rothwell J. C. Caljouw S. R. (2018). The role of task difficulty in learning a visuomotor skill. Med. Sci. Sports Exerc. 50, 1842–1849. doi: 10.1249/MSS.0000000000001635, 29634641

[ref8] Button C. Seifert L. Chow J. Y. Araújo D. Davids K. (2021). Dynamics of Skill Acquisition: An Ecological Dynamics Approach. Champaign, IL: Human Kinetics.

[ref9] Cohen J. (2013). Statistical Power Analysis for the Behavioral Sciences. London: Routledge.

[ref10] Crocker L. Algina J. (1986). Introduction to Classical and Modern Test Theory. New York: Holt, Rinehart and Winston.

[ref11] Davids K. Button C. Bennett S. (2008). Dynamics of Skill Acquisition: A Constraints-Led Approach. Champaign, IL: Human Kinetics.

[ref12] De Waard D. (1996). The Measurement of Drivers' Mental Workload. Groningen: University of Groningen.

[ref13] Ericsson K. A. Krampe R. T. Tesch-Römer C. (1993). The role of deliberate practice in the acquisition of expert performance. Psychol. Rev. 100, 363–406.

[ref14] Farrow D. Abernethy B. Jackson R. C. (2005). Probing expert anticipation with the temporal occlusion paradigm: experimental investigations of some methodological issues. Mot. Control. 9, 330–349. doi: 10.1123/mcj.9.3.330, 16239719

[ref15] Farrow D. Reid M. (2010). The effect of equipment scaling on the skill acquisition of beginning tennis players. J. Sports Sci. 28, 723–732. doi: 10.1080/02640411003770238, 20480427

[ref16] Faul F. Erdfelder E. Lang A. G. Buchner A. (2007). G*power 3: a flexible statistical power analysis program for the social, behavioral, and biomedical sciences. Behav. Res. Methods 39, 175–191. doi: 10.3758/BF03193146, 17695343

[ref17] Fitts P. M. (1954). The information capacity of the human motor system in controlling the amplitude of movement. J. Exp. Psychol. 47:381. 13174710

[ref18] Folland J. P. Williams A. G. (2007). Morphological and neurological contributions to increased strength. Sports Med. 37, 145–168. doi: 10.2165/00007256-200737020-00004 17241104

[ref19] Galle S. Malcolm P. Collins S. H. De Clercq D. (2017). Reducing the metabolic cost of walking with an ankle exoskeleton: interaction between actuation timing and power. J. Neuroeng. Rehabil. 14:35. doi: 10.1186/s12984-017-0235-0, 28449684 PMC5408443

[ref20] Gentile A. M. (2000). Skill acquisition: action, movement, and neuromotor processes. Movement Sci., 111–187.

[ref21] Glencross D. J. Gould J. H. (1979). The planning of precision movements. J. Mot. Behav. 11, 1–9. 15186967 10.1080/00222895.1979.10735167

[ref22] Grundy J. G. Barker R. M. Anderson J. A. E. Shedden J. M. (2019). The relation between brain signal complexity and task difficulty on an executive function task. NeuroImage 198, 104–113. doi: 10.1016/j.neuroimage.2019.05.045, 31112787

[ref23] Guadagnoli M. A. Lee T. D. (2004). Challenge point: a framework for conceptualizing the effects of various practice conditions in motor learning. J. Mot. Behav. 36, 212–224. doi: 10.3200/JMBR.36.2.212-224, 15130871

[ref24] Horak F. B. (2006). Postural orientation and equilibrium: what do we need to know about neural control of balance to prevent falls? Age Ageing 35, ii7–ii11. doi: 10.1093/ageing/afl077, 16926210

[ref25] ITTF (2010). The International Table Tennis Federation Technical Leaflet T4: Racket Coverings. Lausanne: ITTF.

[ref26] ITTF (2025). The International Table Tennis Federation Handbook. Lausanne: ITTF.

[ref27] Kahneman D. (1973). Attention and Effort. Englewood Cliffs, NJ: Prentice-Hall.

[ref28] Kantowitz B. H. (1987). Mental workload. Adv. Psychol. 47, 81–121.

[ref29] Kleim JA Jones TA (2008) Principles of experience-dependent neural plasticity: implications for rehabilitation after brain damage. Journal of Speech, Language, and Hearing Research, 51, S225–S239. doi: 10.1044/1092-4388(2008/018) 18230848

[ref30] Kwakkel G. Van Wegen E. E. Burridge J. H. Winstein C. J. Van Dokkum L. E. H. Alt Murphy M. . (2019). Standardized measurement of quality of upper limb movement after stroke: consensus-based core recommendations from the second stroke recovery and rehabilitation roundtable. Int. J. Stroke 14, 783–791. doi: 10.1177/1747493019873519, 31510885

[ref31] Laffaye G. Phomsoupha M. Dor F. (2015). Changes in the game characteristics of a badminton match: a longitudinal study through the Olympic game finals analysis in men's singles. J. Sports Sci. Med. 14, 584–590. 26335338 PMC4541123

[ref32] Lakens D. (2013). Calculating and reporting effect sizes to facilitate cumulative science: a practical primer for t-tests and ANOVAs. Front. Psychol. 4:863. doi: 10.3389/fpsyg.2013.00863, 24324449 PMC3840331

[ref33] Li Y. Wright D. L. (2000). An assessment of the attention demands during random- and blocked-practice schedules. Q. J. Experimental Psychol. Section A. 53, 591–606. 10.1080/713755890 10881620

[ref34] Liu W. Zhou C. Ji L. Watson J. C. 2nd (2012). The effect of goal setting difficulty on serving success in table tennis and the mediating mechanism of self-regulation. Human Kinetics. 33, 173–185. doi: 10.2478/v10078-012-0056-y, 23487526 PMC3588684

[ref35] Lohse K. R. Sherwood D. E. Healy A. F. (2010). How changing the focus of attention affects performance, kinematics, and electromyography in dart throwing. Hum. Mov. Sci. 29, 542–555. doi: 10.1016/j.humov.2010.05.001, 20541275

[ref36] Marteniuk RG (1976) Information Processing in Motor Skills. New York: Holt, Rinehart and Winston.

[ref37] McAfee R. (2009). Table Tennis: Steps to Success. Champaign, IL: Human Kinetics.

[ref38] Moray N. (1967). Where is capacity limited? A survey and a model. Acta Psychol. 27, 84–92. 10.1016/0001-6918(67)90048-0 6062244

[ref39] National Education Examinations Authority (1991). Theory and Practice of Item Bank Construction. Beijing: Guangming Daily Press.

[ref40] Padulo J. Pizzolato F. Tosi Rodrigues S. Migliaccio G. M. Attene G. Curcio R. . (2016). Task complexity reveals expertise of table tennis players. J. Sports Med. Phys. Fitness 56, 149–156. 25611083

[ref41] Parasuraman R. Hancock P. A. (2000). “Adaptive control of mental workload,” in Stress, Workload, and Fatigue, (Boca Raton, FL: CRC Press), 305–320.

[ref42] Phomsoupha M. Laffaye G. (2015). The science of badminton: game characteristics, anthropometry, physiology, visual fitness and biomechanics. Sports Med. 45, 473–495. doi: 10.1007/s40279-014-0287-2, 25549780

[ref43] Salmoni A. W. Sullivan S. J. Starkes J. L. (1976). The attention demands of movements: a critique of the probe technique. J. Mot. Behav. 8, 161–169. 23964571 10.1080/00222895.1976.10735068

[ref44] Sanli E. A. Lee T. D. (2015). Nominal and functional task difficulty in skill acquisition: effects on performance in two tests of transfer. Hum. Mov. Sci. 41, 218–229. doi: 10.1016/j.humov.2015.03.006, 25846951

[ref45] Schmidt R. A. Lee T. D. Winstein C. Wulf G. Zelaznik H. N. (2018). Motor Control and Learning: A Behavioral Emphasis. Champaign, IL: Human Kinetics.

[ref46] Sternad D. (2018). It's not (only) the mean that matters: variability, noise and exploration in skill learning. Curr. Opin. Behav. Sci. 20, 183–195. doi: 10.1016/j.cobeha.2018.01.004, 30035207 PMC6051545

[ref47] Taghi M. M. Aghdaei M. Farsi A. Badicu G. de Sousa Fernans M. S. Yagin F. H. . (2025). The effect of task cognitive difficulty on perceptual-cognitive indicators: evidence on the relationship between challenge point framework (CPF) and cognitive development in table tennis beginners. J. Multidiscip. Healthc. 18, 407–419. doi: 10.2147/JMDH.S472671, 39881822 PMC11776927

[ref48] Ting L. H. McKay J. L. (2007). Neuromechanics of muscle synergies for posture and movement. Curr. Opin. Neurobiol. 17, 622–628. doi: 10.1016/j.conb.2008.01.002, 18304801 PMC4350235

[ref49] Wang B. C. (2021). Physical Training Evaluation Methods and Applications. Tianjin: Tianjin Academy of Social Sciences Press.

[ref50] Wang LY “Research on speed, spin, placement and tactical game in table tennis.” In: *Proceedings of the 13th National Sports Science Conference*. Beijing: China Sport Science Society; (2023).

[ref51] Wolpert D. M. Ghahramani Z. (2000). Computational principles of movement neuroscience. Nat. Neurosci. 3, 1212–1217. 11127840 10.1038/81497

[ref52] Wulf G. Shea C. H. (2002). Principles derived from the study of simple skills do not generalize to complex skill learning. Psychon. Bull. Rev. 9, 185–211. doi: 10.3758/BF03196276, 12120783

[ref53] Xie W Teh KC Qin ZF “Speed and spin of 40mm table tennis ball and the effects on elite players.” In: *ISBS - Conference Proceedings Archive*. International Society of Biomechanics in Sports; (2008).

[ref54] Zhang H. C. (2005). Practical Psychological Assessment. Beijing: China Light Industry Press, 80–85.

